# Polymer-Dependent Morphology Development of Biodegradable Porous Microspheres with Amphiphilic PEG-PDLLA Incorporation

**DOI:** 10.3390/polym18192415

**Published:** 2026-10-02

**Authors:** Jin Cheol Cho, Sun Woo Jung, Gunpoong Kim

**Affiliations:** R&D Center, VAIM Co., Ltd., 30, Techno 1-ro, Yuseong-gu, Daejeon 34016, Republic of Korea; swjung@vaim.co.kr (S.W.J.); gunpkim@vaim.co.kr (G.K.)

**Keywords:** biodegradable porous microspheres, PEG-PDLLA, polymer properties, porous structure formation, amphiphilic polymer, injectable biomaterials, tissue engineering, regenerative fillers

## Abstract

Biodegradable porous microspheres hold considerable promise as regenerative fillers, injectable biomaterials, tissue-engineering scaffolds, and drug-delivery carriers; however, how polymer properties and amphiphilic modification jointly influence pore formation remains insufficiently understood. In this study, porous microspheres composed of poly(D,L-lactide) (PDLLA), poly(L-lactide) (PLLA), poly(ε-caprolactone) (PCL), and amphiphilic PEG-PDLLA were fabricated by a porogen-assisted solvent evaporation method, with PEG-PDLLA incorporated as an amphiphilic morphology-modifying polymer component. The relationships among polymer type and molecular weight, particle size, porogen incorporation, PEG-PDLLA incorporation, and microsphere morphology were evaluated using scanning electron microscopy, tapped-density porosity analysis, laser diffraction, Brunauer–Emmett–Teller (BET) surface area analysis, micro-computed tomography (Micro-CT), and differential scanning calorimetry (DSC). PDLLA exhibited the highest corrected density-derived porosity index (42.4%), followed by PLLA (30.9%) and PCL (13.6%). Smaller PDLLA microspheres showed higher corrected density-derived porosity, reaching 50.5% at a median particle diameter (D_50_) of 28.7 μm. PEG-PDLLA incorporation was associated with the formation of surface-open pores and heterogeneous internal architectures as well as increased nitrogen-accessible BET surface area and pore volume, with response exhibited in a polymer-dependent manner. Micro-CT showed differences in internal density distribution and architecture between PLLA and PLLA/PEG-PDLLA microspheres, while DSC revealed polymer-dependent thermal characteristics without fundamentally altering the characteristic thermal transitions of the host polymers. Overall, porous microsphere formation was associated with polymer physicochemical properties, PEG-PDLLA-associated amphiphilic morphology modification, molecular weight, particle size, porogen incorporation, and emulsion/solidification behavior. These findings provide a comparative framework for investigating formulation strategies for biodegradable porous microspheres with tunable architecture and surface accessibility for regenerative medicine and controlled drug-delivery applications.

## 1. Introduction

Biodegradable polymer microspheres have attracted considerable attention in regenerative medicine, tissue engineering, drug delivery, and aesthetic medicine because of their excellent biocompatibility, biodegradability, and tunable physicochemical properties [1,2,3,4,5]. Injectable biodegradable microspheres have been extensively utilized as regenerative fillers, collagen stimulators, tissue scaffolds, and cell-interactive biomaterials because they provide temporary structural support while promoting extracellular matrix (ECM) remodeling and tissue regeneration [3,4,5,6,7,8]. Recent advances in regenerative biomaterials have highlighted the importance of precisely controlling microsphere morphology, pore architecture, and surface characteristics to optimize cell–material interactions, tissue integration, degradation behavior, and therapeutic performance [2,3,4,5,6,7,8].

Among biodegradable polymers, poly(D,L-lactide) (PDLLA), poly(L-lactide) (PLLA), and poly(ε-caprolactone) (PCL) are widely used owing to their established biocompatibility and controllable degradation characteristics [6,9,10,11,12,13,14]. However, these polymers exhibit markedly different physicochemical properties, including crystallinity, molecular mobility, and solidification behavior, which may contribute to differences in microsphere formation and pore stabilization [6,10,11,13,15]. PDLLA is an amorphous polymer with chain-packing and solidification characteristics that may favor pore development under appropriate solvent-evaporation conditions [10,11,13,15,16]. In contrast, PLLA and PCL are semicrystalline polymers with different crystallization kinetics and mechanical stability, resulting in distinct porous morphologies and degradation behaviors [9,10,13,15]. Understanding how these intrinsic polymer properties are associated with pore development, pore stabilization, and porous microsphere morphology is therefore essential for the rational design of biodegradable regenerative biomaterials [6,12,13,15,16].

Porous biodegradable microspheres have received increasing attention because their internal architecture and surface-open pore structures strongly influence biological responses [3,4,17,18,19,20,21,22]. Interconnected porous networks increase the available surface area, facilitate cell attachment and migration, improve nutrient transport, and promote tissue infiltration and ECM deposition [5,17,18,19,20,21,22]. In addition, pore architecture affects hydration behavior, degradation kinetics, and drug-release characteristics, making pore control a key design parameter for regenerative fillers, injectable biomaterials, and tissue-engineering scaffolds [2,3,4,17,18,19,20,21,22].

Various fabrication methods have been developed to prepare porous biodegradable microspheres, including solvent evaporation, solvent extraction, double emulsion, spray drying, thermally induced phase separation, and porogen-assisted techniques [16,23,24,25,26]. Among these approaches, porogen-assisted solvent evaporation is particularly attractive because it simultaneously enables particle formation and pore generation under relatively simple processing conditions [23,24,26,27,28]. Nevertheless, pore formation remains difficult to control because it can be influenced by multiple interconnected factors, including polymer crystallinity, molecular weight, solvent exchange kinetics, emulsion droplet stability, and porogen extraction [15,16,23,24,25,26,28]. Accordingly, the selection of fabrication conditions can directly influence particle formation, pore development, and the preservation of the resulting porous architecture. Thus, optimization of porous microsphere design requires consideration of both material properties and processing conditions rather than porogen incorporation alone. Excessive pore generation may cause droplet collapse, whereas insufficient pore formation results in dense microspheres with limited biological functionality [24,25,26,27,28]. Consequently, a better understanding of polymer-dependent morphology development during porous microsphere fabrication remains an important challenge for the rational design of regenerative biomaterials [15,16,22,23,24,25,26].

Recently, amphiphilic block copolymers have emerged as effective morphology modifiers because they can influence interfacial properties, solvent exchange, and phase separation during particle formation, and PEG-based amphiphilic copolymers in particular have shown potential to modify polymer matrices through polymer redistribution and phase-separation-related processes, thereby altering pore architecture and improving surface accessibility [24,25,26,27,28,29,30,31,32,33,34]. For example, amphiphilic mPEG-PLA and mPEG-PLGA have been used to prepare porous microspheres without conventional porogens, and differences in their hydrophobic polymer segments were shown to alter solvent affinity, phase-separation behavior, and the resulting internal morphology [34]. In parallel, ammonium-bicarbonate-assisted fabrication of porous PLGA microspheres has demonstrated that gas-generating porogens can influence both emulsion behavior and interconnected pore formation during solvent evaporation [27,28]. These effects are expected to depend on the chemical characteristics and composition of the host polymer, as well as the relative composition of the amphiphilic copolymer. Although PEG-containing copolymers have been extensively investigated for drug delivery and biomaterials, their role in controlling the morphology of biodegradable porous microspheres, as well as their interactions with different biodegradable host polymers, have received comparatively little attention [16,24,26,29,30,31,32,33,34,35]. In particular, systematic comparisons applying the same amphiphilic morphology-modifying component across host polymers with distinct molecular and crystalline characteristics remain limited [15,16,26,34].

In addition to polymer composition, particle size and molecular weight are important but often overlooked parameters associated with porous microsphere formation [11,13,15,24,26]. Particle size influences solvent diffusion, solidification kinetics, and pore preservation, whereas polymer molecular weight influences chain entanglement and emulsion behavior during emulsification [11,13,15,23,24,26]. These processing–material interactions can ultimately affect measurable structural characteristics, including particle size distribution, porosity, surface area, and pore accessibility. However, systematic investigations into the combined relationships among particle size fractionation, molecular weight, polymer characteristics, and amphiphilic polymer incorporation in porous microsphere morphology are still limited [11,15,16,23,24,25,26,29,30,31,32,33,34,36].

Another challenge lies in the characterization of porous structures. Conventional density-based porosity measurements reflect both interparticle voids and intraparticle pores, and therefore cannot directly quantify intraparticle pore volume without considering particle packing and interparticle void contributions [18]. Therefore, complementary analytical techniques—including scanning electron microscopy (SEM), micro-computed tomography (Micro-CT), Brunauer–Emmett–Teller (BET) surface area analysis, and differential scanning calorimetry (DSC)—are required to comprehensively evaluate surface morphology, internal architecture, pore accessibility, and thermal behavior. In particular, BET surface area and gas-adsorption-based pore analysis provide quantitative information on the nitrogen-accessible surface area and pore structure [37,38]. The integration of these complementary approaches provides a more complete picture of morphology development than any single technique alone.

In the present study, biodegradable porous composite microspheres composed of PDLLA, PLLA, PCL, and amphiphilic PEG-PDLLA were fabricated using a porogen-assisted solvent evaporation method. The relationships among polymer type and molecular weight, particle size fractionation, porogen incorporation, PEG-PDLLA incorporation, and microsphere morphology were systematically investigated using SEM, tapped-density-based porosity analysis, laser diffraction, Micro-CT, BET, and DSC.

In contrast to previous studies focusing primarily on individual polymer systems or specific pore-forming approaches [15,16,26,27,28,34,36], the present study applies the same PEG-PDLLA morphology-modifying polymer component across PDLLA, PLLA, and PCL matrices to compare porous morphology development among different host polymers under a common experimental framework.

Based on these complementary analyses, this study examines polymer-dependent morphological trends associated with polymer properties, PEG-PDLLA incorporation, molecular weight, particle size, porogen incorporation, and emulsion/solidification conditions. These findings provide a comparative basis for further investigation of formulation strategies for biodegradable porous microspheres intended for regenerative fillers, injectable biomaterials, tissue-engineering scaffolds, cell-interactive carriers, and drug-delivery systems.

## 2. Materials and Methods

### 2.1. Materials

Poly(D,L-lactide) (PDLLA; RESOMER^®^ R202S, R205S, and R207S), poly(L-lactide) (PLLA; RESOMER^®^ L206S), and poly(ε-caprolactone) (PCL; RESOMER^®^ C209) were purchased from Evonik Industries AG (Essen, Germany). Methoxy poly(ethylene glycol)-block-poly(D,L-lactide) (mPEG-DLPLA; product code DL1005MP1; hereafter referred to as PEG-PDLLA) was purchased from Nomisna Healthcare Pvt. Ltd (Vadodra, India). The commercial polymers and their physicochemical characteristics are summarized in Table 1.

Poly(vinyl alcohol) (PVA; CAS No. 9002-89-5; degree of polymerization (DP) = 500; 87–89% hydrolyzed; Daejung Chemicals & Metals Co., Ltd., Siheung, Republic of Korea) was used as an emulsifying stabilizer. Ammonium bicarbonate (NH_4_HCO_3_; Duksan Pure Chemicals Co., Ltd., Ansan, Republic of Korea) was used as the porogen. Dichloromethane (DCM; HPLC grade; Duksan Pure Chemicals Co., Ltd., Ansan, Republic of Korea) was used as the organic solvent. All chemicals were used as received without further purification.

### 2.2. Preparation of Microspheres

#### 2.2.1. Non-Porous Microspheres

The preparation procedure is schematically illustrated in Figure 1A. Non-porous microspheres were prepared by a conventional oil-in-water (O/W) solvent evaporation method. Biodegradable polymers (PDLLA, PLLA, or PCL) were dissolved in dichloromethane (DCM) at a polymer concentration of approximately 5 wt% to prepare the organic phase. No PEG-PDLLA or porogen was added. The organic phase was emulsified in an aqueous poly(vinyl alcohol) (PVA, 0.5–1.0 wt%) solution under mechanical stirring (approximately 800 rpm). The emulsion was stirred for approximately 6 h at room temperature to allow solvent evaporation and microsphere solidification. The microspheres were collected by filtration and washed repeatedly with distilled water. Prior to lyophilization, the microspheres were frozen at −40 °C in a deep freezer until completely frozen. The frozen samples were then lyophilized for 48 h using a freeze dryer (FD8512, Ilshin BioBase Co., Ltd., Dongducheon, Gyeonggi-do, Republic of Korea) with a condenser temperature of approximately −80 °C and a chamber pressure of approximately 5 mTorr. These samples, prepared without porogen incorporation, were used as reference microspheres for calculation of the corrected density-derived porosity index described in Section 2.6.

#### 2.2.2. Porogen-Assisted Porous Microspheres

The preparation procedure is illustrated in Figure 1B. Porous microspheres were fabricated using a porogen-assisted water-in-oil-in-water (W/O/W) solvent-evaporation method. Biodegradable polymers were dissolved in DCM to form the organic phase. A 10 wt% aqueous NH_4_HCO_3_ solution was added at an aqueous-to-organic phase volume ratio of approximately 1:39 (*v*/*v*), followed by ultrasonication to form a primary W/O emulsion. The primary emulsion was subsequently dispersed into an aqueous PVA solution to form a W/O/W emulsion. The resulting emulsion was mechanically stirred for approximately 6 h to allow solvent evaporation and microsphere solidification. The microspheres were then collected by filtration, thoroughly washed with distilled water to remove residual PVA and NH_4_HCO_3_, and freeze-dried under the same conditions described in Section 2.2.1 prior to further characterization.

#### 2.2.3. PEG-PDLLA Composite Microspheres

PEG-PDLLA composite microspheres were prepared as illustrated in Figure 1C,D. PEG-PDLLA was blended with the biodegradable polymer at predetermined weight ratios prior to emulsification. For porogen-assisted formulations (Figure 1C), a 10 wt% aqueous NH_4_HCO_3_ solution was incorporated into the PEG-PDLLA/polymer solution and dispersed by ultrasonication to form a W/O emulsion. The resulting primary emulsion was subsequently dispersed in an aqueous PVA solution to form a W/O/W emulsion. For porogen-free formulations (Figure 1D), PEG-PDLLA was directly dissolved together with the biodegradable polymer, and microspheres were prepared using an O/W solvent-evaporation method without NH_4_HCO_3_. In both formulations, the emulsions were mechanically stirred for approximately 6 h to allow solvent evaporation and microsphere solidification. The resulting microspheres were collected by filtration, repeatedly washed with distilled water, and freeze-dried under the same conditions described in Section 2.2.1 prior to further characterization.

### 2.3. Particle Size Fractionation

Particle size fractionation was performed using a custom-built suspended planar mesh sieving apparatus, in which microspheres were separated under wet conditions to minimize mechanical damage to the porous structure. The microspheres were dispersed in distilled water and passed through suspended stainless-steel mesh screens to obtain three particle size fractions (<33 μm, 33–60 μm, and >60 μm). The fractionated microspheres were collected by filtration, freeze-dried under the same conditions described in Section 2.2.1, and subsequently characterized to compare pore morphology and the corrected density-derived porosity index among the different particle size fractions.

### 2.4. Particle Size Analysis

Particle size distributions were measured using a laser diffraction particle size analyzer (LA-960, Horiba Ltd., Kyoto, Japan). Prior to analysis, the microspheres were dispersed in distilled water and measured under continuous stirring to minimize particle sedimentation during analysis. Particle size distributions were expressed as D_10_, D_50_, and D_90_ values, and the Span value was used to characterize the width of the particle size distribution. The median particle diameter (D_50_) was used as the representative particle size. The Span, representing the width of the particle size distribution, was calculated according to the following equation:

(1) $$\begin{array}{cccc} & Span=\frac{D_{90}-D_{10}}{D_{50}} & & \end{array}$$

where D_10_, D_50_, and D_90_ represent the particle diameters at 10%, 50%, and 90% cumulative volume, respectively.

### 2.5. Scanning Electron Microscopy (SEM)

The surface morphology and cross-sectional pore structure of the microspheres were examined using scanning electron microscopy (SEM; PE-100, ModuleSci Co., Ltd., Daejeon, Republic of Korea). The microspheres were mounted on aluminum stubs using conductive carbon tape and sputter-coated with gold prior to observation. For cross-sectional observation, the microspheres were immobilized in a high-molecular-weight hyaluronic acid (HA) matrix (approximately 3 MDa), air-dried, and sectioned using a razor blade (ST300, Dorco Co., Ltd., Seoul, Republic of Korea). This embedding procedure was used to minimize particle displacement during sectioning and to facilitate observation of the internal pore architecture. Representative SEM images were obtained to qualitatively compare surface morphology, surface pore openings, internal pore architecture, and overall particle shape among the different polymer systems and preparation conditions.

### 2.6. Porosity Measurement and Calculation

The porosity of the microspheres was estimated from tapped-density measurements. Tapped density was determined after 1250 tapping cycles using a tapped density analyzer (BeDensi T1 Pro, Bettersize Instruments Ltd., Dandong, China). All measurements were performed in triplicate, and the average value was used for subsequent porosity calculations.

The apparent porosity (*P*_app_) was calculated according to the following equation [18,22]:

(2) $$P_{app}\left(\%\right)=\left(1-\frac{\rho_{tap}}{\rho_{ref}}\right)\times 100$$

where *ρ*_*tap*_ is the experimentally measured tapped density of the microspheres and *ρ*_*r*ef_ is the literature-based reference density of the corresponding polymer or polymer composition. The reference density values used were assumed to be 1.25, 1.27, 1.145, and 1.22 g cm^−3^ for PDLLA, PLLA, PCL, and PEG–PDLLA, respectively. For composite formulations, the reference density was estimated from the densities of the constituent polymers according to their formulation ratio. These values were not sample-specific skeletal densities measured by gas pycnometry.

Because *P*_app_ includes both intraparticle pores and interparticle voids, a corrected density-derived porosity index (*P*_corr_) was estimated for the pure-polymer systems by subtracting the apparent porosity of the corresponding non-porous microspheres:

(3) $$\begin{array}{cccc} & P_{corr}\left(\%\right)=P_{app,porous}-P_{app,nonporous} & & \end{array}$$

This reference-based correction partially accounts for interparticle packing effects but does not directly measure intraparticle pore volume. Therefore, *P*_corr_ was used as a density-derived comparative index rather than as a direct measure of absolute intraparticle porosity.

For PEG-PDLLA-containing composites, the porogen-free formulation could not be considered non-porous because porous morphology was also observed in the absence of the conventional porogen. Therefore, porogen-induced incremental porosity (*P*_inc_) was defined as:

(4) $$\begin{array}{cccc} & P_{inc}\left(\%\right)=P_{app,porogen-assisted}-P_{app,porogen-free} & & \end{array}$$

*P*_inc_ represents the incremental difference in the apparent porosity associated with ammonium bicarbonate incorporation and not the total porosity of the composite microspheres. Accordingly, *P*_corr_ and *P*_inc_ were treated as distinct parameters and were not directly compared as equivalent measures of total intraparticle pore volume.

### 2.7. BET Surface Area Analysis

The specific surface area and nitrogen-accessible pore characteristics of the microspheres were analyzed using a surface area and porosity analyzer (TriStar™ II 3020, Micromeritics Instrument Corporation, Norcross, GA, USA). Before analysis, the samples were degassed under vacuum to remove physically adsorbed moisture and volatile contaminants. Nitrogen adsorption–desorption isotherms were obtained at 77 K using high-purity nitrogen gas. The specific surface area was calculated using the Brunauer–Emmett–Teller (BET) method. The BJH adsorption pore volume and average pore diameter were determined using the Barrett–Joyner–Halenda (BJH) adsorption model, while the micropore surface area and external surface area were evaluated using the t-plot method.

### 2.8. Differential Scanning Calorimetry (DSC)

Thermal properties of the microspheres were analyzed using a differential scanning calorimeter (DSC 823e, Mettler Toledo, Greifensee, Switzerland) under a nitrogen atmosphere. Approximately 5–10 mg of each sample was sealed in an aluminum pan for analysis.

For PCL-containing samples, a heat–cool–heat program was employed to eliminate thermal history and evaluate crystallization behavior. The samples were heated from −50 °C to 220 °C, cooled to −50 °C, and reheated to 220 °C at a heating and cooling rate of 10 °C min^−1^.

For PDLLA- and PLLA-containing samples, DSC measurements were performed from 0 °C to 220 °C at a heating rate of 10 °C min^−1^. For samples evaluated using a heat–cool–heat program, the same heating and cooling rate of 10 °C min^−1^ was applied.

Representative thermal transition temperatures were selected according to the characteristic thermal behavior of each polymer system. Because PDLLA is an amorphous polymer, the T_g_-related endothermic transition was used as the representative thermal transition in the absence of a distinct polymer melting peak. For the semicrystalline polymers (PLLA and PCL), the representative melting temperature (T_m_) was obtained from the second heating scan when a heat–cool–heat program was applied after the removal of thermal history. In contrast, the characteristic melting transition of PEG-PDLLA was determined from the first heating scan because no distinct recrystallization peak was observed during the subsequent cooling and reheating cycle.

### 2.9. Micro-Computed Tomography (Micro-CT)

The internal architecture of the microspheres was examined using a micro-computed tomography (Micro-CT) system (SkyScan 1272, Bruker AXS, Kontich, Belgium). Two-dimensional (2D) cross-sectional images and three-dimensional (3D) reconstructed images were acquired to visualize the internal pore architecture and density distribution within the microspheres.

Qualitative comparisons were performed between PLLA microspheres and PLLA/PEG-PDLLA composite microspheres to examine differences in internal pore morphology and structural organization associated with PEG-PDLLA incorporation.

### 2.10. Statistical Analysis

Quantitative data were analyzed primarily using descriptive comparisons. Repeated measurements were performed for selected representative samples, including particle-size and tapped-density analyses, to assess analytical repeatability. These measurements were treated as technical replicates and did not represent independently prepared formulation batches.

Because independent replicate batches were not available for all formulations, formal inferential statistical analyses, including ANOVA and post hoc multiple-comparison tests, were not performed. Accordingly, quantitative differences among formulations are presented and interpreted as descriptive experimental trends rather than statistically significant differences.

### 2.11. Use of Generative Artificial Intelligence

During the preparation of this manuscript, the authors used ChatGPT (OpenAI, GPT-5.6) for language refinement and improvement of the clarity and readability of the manuscript. The tool was not used for experimental design, data generation, or independent scientific interpretation of the experimental results. All AI-assisted content was critically reviewed and edited by the authors, who take full responsibility for the final content of the manuscript.

## 3. Results and Discussion

### 3.1. Morphology and Particle Size Distribution of As-Prepared PDLLA Microspheres

Porous PDLLA microspheres were successfully fabricated by the porogen-assisted solvent evaporation method. As shown in Figure 2a, the as-prepared microspheres exhibited a broad particle size distribution with D_10_, D_50_, and D_90_ values of 36.2, 68.2, and 125.3 μm, respectively, corresponding to a Span value of 1.31. The broad size distribution is consistent with the formation of emulsion droplets with different diameters during emulsification, followed by solvent evaporation and particle solidification [23,24].

Representative SEM images (Figure 2b–d) showed that the microspheres possessed a well-defined spherical morphology with numerous surface-open pores distributed over the particle surface.

At higher magnification, circular pore openings of varying sizes were clearly observed over the particle surface, indicating successful pore formation while preserving the overall spherical morphology of the microspheres. No apparent collapse, cracking, or severe deformation was observed, indicating that the porous surface morphology and overall particle shape were preserved under the investigated preparation conditions.

The broad particle size distribution observed in the as-prepared microspheres indicated the need to evaluate whether porous morphology and density-derived porosity characteristics differed among particle size fractions. Therefore, particle size fractionation was subsequently performed to investigate the relationship between particle size, pore morphology, and the corrected density-derived porosity index.

### 3.2. Microsphere Morphology and Density-Derived Porosity According to Particle Size Fractionation

Porous PDLLA (RESOMER^®^ R207S) microspheres were separated into three particle size fractions using a custom-built suspended planar mesh fractionation apparatus. Particle size fractionation produced three distinct microsphere populations with D_50_ values of 28.7, 46.6, and 84.3 μm, corresponding to the <33 μm, 33–60 μm, and >60 μm fractions, respectively (Table 2). Compared with the unfractionated microspheres (Span = 1.31), fractionation substantially narrowed the particle size distribution, yielding Span values of 0.80, 0.57, and 0.65, respectively. Among the three fractions, the 33–60 μm fraction exhibited the narrowest particle size distribution.

Representative SEM images (Figure 3) showed that all size fractions retained well-defined spherical morphology with uniformly distributed surface-open pores after fractionation. No noticeable particle deformation, fracture, or pore collapse was observed, indicating that the suspended planar mesh fractionation process effectively preserved the observed porous surface morphology and overall particle shape.

The corrected density-derived porosity index gradually decreased with increasing particle size, from 50.5% in the <33 μm fraction to 42.4% and 41.9% in the 33–60 μm and >60 μm fractions, respectively (Table 2).

The higher corrected density-derived porosity index observed in the smallest microspheres may be attributed to their larger surface-area-to-volume ratio, which may facilitate solvent exchange and contribute to earlier solidification during solvent evaporation [23,24]. Although solvent diffusion and solidification kinetics were not directly measured in the present study, this interpretation is consistent with the possibility that smaller microspheres undergo more rapid solvent removal relative to their volume, which may favor preservation of pore features before substantial structural rearrangement occurs. In contrast, larger microspheres may undergo slower solvent removal, potentially allowing greater polymer chain mobility and pore rearrangement before complete solidification.

Although the medium- and large-sized fractions exhibited similar corrected density-derived porosity indices, the relatively small difference between the 33–60 μm and >60 μm fractions suggests that the particle-size association with the density-derived porosity index may become less pronounced beyond a certain size range under the present processing conditions. This apparent plateau-like trend suggests that the relationship between particle size and the density-derived porosity index may not be linear and may also be influenced by solvent removal, polymer solidification, and particle packing effects. Nevertheless, the consistently higher density-derived porosity index of the smallest fraction suggests an association between particle size fraction and the preservation of porous morphology, particularly in the smaller particle-size range. These results indicate that particle size fractionation not only improves particle size uniformity but also enables the selection of porous microsphere populations with distinct density-derived porosity characteristics. Collectively, these findings suggest that particle size is a controllable processing parameter associated with differences in porous morphology and density-derived porosity characteristics during solvent evaporation and subsequent microsphere solidification.

### 3.3. Effect of Polymer Type and Molecular Weight on Porous Microsphere Formation

To investigate the effects of polymer type and molecular weight on porous microsphere formation, porous and corresponding porogen-free microspheres prepared from PLLA, PDLLA with different molecular weights, and PCL were compared under identical fabrication conditions (Table 3). Representative SEM images of the selected polymer systems are shown in Figure 4.

Among the PDLLA grades, the corrected density-derived porosity index increased markedly with molecular weight, rising from 16.9% for R202S (16 kDa) to 30.7% for R205S (63 kDa) and reaching 42.4% for R207S (190 kDa) (Table 3). A similar trend was observed for particle size, with the D_50_ increasing from 39.3 μm to 47.5 μm and 68.1 μm, respectively. The increase in particle size with molecular weight may be associated with increased polymer-solution viscosity and chain entanglement, which can influence droplet breakup during emulsification [11,13,23,24]. In addition, the broader particle size distribution observed for R207S (Span = 1.31) suggests that highly entangled polymer chains may contribute to the formation of droplets with a wider range of diameters during emulsification. These results suggest that molecular weight may influence emulsion droplet behavior and preservation of the porous structure during solvent evaporation.

The influence of polymer type was further evaluated by comparing PLLA, PDLLA (R207S), and PCL microspheres prepared using the porogen-assisted process (Figure 4a–f). Although all three polymers produced spherical microspheres with porous surfaces, their internal pore architectures differed markedly. Cross-sectional SEM images revealed that amorphous PDLLA exhibited extensive internal porous features throughout the particle interior (Figure 4d). In contrast, PLLA exhibited a moderately porous internal structure while maintaining spherical morphology (Figure 4b). PCL showed the lowest corrected density-derived porosity index (13.6%) among the investigated polymers (Table 3), and its cross-sectional image showed a comparatively dense internal structure with limited pore development (Figure 4f).

These differences suggest that polymer-dependent pore formation cannot be attributed to crystallinity alone, but instead may reflect the combined influence of molecular characteristics, chain mobility, crystallinity-related chain packing, and solidification behavior. The amorphous character of PDLLA may be associated with greater chain mobility during solvent evaporation and solidification, whereas the semicrystalline character of PLLA and PCL may contribute to different pore-formation and stabilization behavior during solidification.

The contribution of the porogen was evaluated by comparing PDLLA (R207S) microspheres prepared with and without ammonium bicarbonate (Figure 4c,d,g,h). Both formulations maintained a spherical morphology; however, the internal structures differed substantially. The porogen-assisted PDLLA microspheres exhibited extensive internal porous features (Figure 4d), whereas the porogen-free PDLLA microspheres showed an essentially dense internal structure with limited internal pore development (Figure 4h). These observations indicate that ammonium bicarbonate was associated with substantial internal pore development in the PDLLA system under the present processing conditions. The characteristics of the amorphous PDLLA matrix may also have contributed to the preservation of the resulting porous morphology during solvent evaporation and solidification.

Overall, the results suggest that porous microsphere formation is influenced by multiple polymer and processing factors, including molecular weight, chain mobility, crystallinity-related chain packing, and porogen-assisted pore formation. Higher molecular weight may improve droplet stability through increased solution viscosity and chain entanglement [11,13,23,24], whereas the amorphous character of PDLLA may provide greater chain mobility during solvent evaporation and solidification [10,11,13]. In contrast, the semicrystalline characteristics of PLLA and PCL may contribute to different chain-packing and solidification behavior under the present processing conditions [9,10,11]. Because crystallinity was not quantitatively determined in the present study, the observed differences in corrected density-derived porosity indices and internal pore architecture should not be attributed to crystallinity alone.

### 3.4. Effect of PEG-PDLLA Incorporation on Polymer-Dependent Pore Formation

The incorporation of PEG-PDLLA influenced porous microsphere formation, and its effect varied according to the physicochemical characteristics of the host polymer (Table 4 and Figure 5). Unlike conventional porogen-assisted pore formation, PEG-PDLLA was associated with amphiphilic morphology modification and porous structure development even in the absence of an internal aqueous porogen. The PDLLA/PEG-PDLLA composites prepared using the higher-molecular-weight PDLLA (R205S) successfully formed spherical porous microspheres under both porogen-assisted and porogen-free conditions.

Representative SEM images of the porogen-free formulation (Figure 5a,b) revealed well-developed surface pores together with internal porous features, indicating that porous morphology could be obtained following PEG-PDLLA incorporation without a conventional porogen. This observation is consistent with possible amphiphilic polymer redistribution or phase-separation-related behavior during solvent evaporation [30,31]. The porogen-induced incremental porosity increased from 12.5% for the 5:5 blend to 15.9% for the 7:3 blend, indicating that reducing the PEG-PDLLA fraction increased the additional pore contribution generated by ammonium bicarbonate while maintaining structural integrity.

In contrast, the low-molecular-weight PDLLA (R202S) system exhibited markedly different behavior. Although spherical porous microspheres were successfully obtained under porogen-assisted conditions (D_50_ = 49.3 μm, Span = 1.13), the corresponding porogen-free formulation produced only irregular aggregates without recoverable spherical particles. These observations suggest that the low molecular weight and associated limited chain entanglement of R202S may have been insufficient to maintain spherical microsphere formation in the absence of an internal aqueous phase [23,24]. The different morphology observed after porogen incorporation suggests that the internal aqueous phase influenced emulsion and solidification behavior during solvent evaporation, although its specific contribution to droplet stability was not directly determined. Thus, within the PDLLA systems investigated, the R202S formulation shows a molecular-weight-associated limitation under porogen-free conditions, although the available molecular-weight levels are insufficient to establish a quantitative molecular-weight threshold.

The PLLA/PEG-PDLLA composites also exhibited successful porous structure formation under both preparation conditions. Representative SEM images (Figure 5c–f) showed uniformly distributed surface-open pores together with internal porous features regardless of porogen incorporation. Notably, the porogen-free PLLA/PEG-PDLLA (5:5) composite exhibited a high apparent porosity of 83.3%, indicating that a highly porous, density-derived structure was obtained following PEG-PDLLA incorporation without a conventional porogen. Following ammonium bicarbonate incorporation, the apparent porosity further increased to 90.1%, suggesting that PEG-PDLLA-associated morphology modification and porogen-assisted pore formation together enhanced pore development. Increasing the PLLA fraction from a 5:5 to a 7:3 blend further increased the porogen-induced incremental porosity from 8.6% to 28.2%, suggesting that an appropriate balance between the semicrystalline PLLA matrix and the amphiphilic copolymer was associated with differences in pore development while maintaining spherical morphology.

A contrasting response was observed for the PCL system. The PCL/PEG-PDLLA (5:5) formulation failed to produce spherical microspheres under porogen-assisted conditions and instead generated irregular aggregates (Table 4), whereas spherical porous microspheres were successfully obtained under porogen-free conditions. Reducing the PEG-PDLLA content to a 7:3 ratio restored microsphere formation under both preparation conditions. Representative SEM images of the porogen-free PCL/PEG-PDLLA (7:3) formulation (Figure 5g) demonstrated the formation of spherical microspheres with surface-open pores, whereas the PEG-PDLLA-rich formulation (Figure 5h) produced irregular collapsed aggregates rather than recoverable microspheres.

These observations suggest that a high PEG-PDLLA fraction alone was insufficient to maintain stable microsphere morphology under the investigated processing conditions and that an appropriate biodegradable polymer matrix was required to support spherical microsphere formation during solvent evaporation. The porogen-induced incremental porosity of the PCL/PEG-PDLLA (7:3) formulation reached 19.8%, indicating that reducing the PEG-PDLLA content restored spherical microsphere formation while maintaining porous morphology.

Accordingly, for the PCL-based system under porogen-assisted conditions, reducing the PEG-PDLLA fraction from 50% (5:5) to 30% (7:3) was associated with a transition from aggregate formation to recoverable spherical microsphere formation under the processing conditions investigated. By comparison, PLLA-based systems maintained spherical morphology over the tested PEG-PDLLA compositions, which may reflect the greater structural stability associated with the semicrystalline PLLA matrix. Taken together, the R202S, PCL, and PLLA results indicate that the practical formulation window depends on the combined effects of host-polymer molecular characteristics, crystallinity-related chain packing, PEG-PDLLA fraction, and porogen-dependent processing conditions rather than on any single material parameter. However, these observations should not be interpreted as universal molecular-weight or crystallinity thresholds, as microsphere formation is also influenced by polymer composition, porogen loading, solvent exchange, and emulsion and solidification conditions.

The PEG-PDLLA-only control provided additional evidence for the role of the biodegradable polymer matrix. Although laser diffraction detected aggregate sizes comparable to those of the composite formulations, SEM analysis revealed only collapsed irregular aggregates without recoverable spherical microspheres. This result indicates that PEG-PDLLA alone did not form recoverable spherical microspheres under the processing conditions investigated supporting its interpretation as an amphiphilic morphology-modifying polymer component whose effect on porous morphology depends on the presence and properties of the host biodegradable polymer.

Overall, PEG-PDLLA was associated not only with pore formation but also with changes in microsphere morphology and pore accessibility in a host-polymer-dependent manner. The observed effects may involve amphiphilic polymer redistribution and phase-separation-related behavior during solvent evaporation; however, these mechanisms were not directly resolved in the present study. The resulting porous architecture was associated with the combined effects of polymer molecular weight, chain mobility, crystallinity-related chain packing, PEG-PDLLA composition, and porogen incorporation. Thus, the present results provide a basis for a polymer-specific formulation strategy involving PEG-PDLLA incorporation and structural support from the host biodegradable polymer matrix.

### 3.5. Surface-Open Pore Morphology and Density-Derived Porosity as Complementary Structural Parameters

One of the key findings of this study is that surface-open pore morphology and density-derived porosity characteristics represent complementary rather than interchangeable structural parameters. Although both parameters describe the porous nature of biodegradable microspheres, they represent different aspects of the pore architecture and therefore provide complementary structural information.

Representative SEM observations demonstrated that PEG-PDLLA-containing composite microspheres, particularly the PLLA/PEG-PDLLA formulations, possessed well-developed surface-open pores together with heterogeneous internal pore features (Figure 5). These pronounced surface and internal pore features were observed even in formulations whose reported porogen-induced incremental porosity (*P*_inc_) represents only the additional apparent porosity associated with ammonium bicarbonate incorporation and does not represent the total porosity of the PEG-PDLLA-containing microspheres. In contrast, PDLLA microspheres exhibited a high corrected density-derived porosity index (*P*_corr_) relative to their non-porous reference formulation, but a comparatively lower density of surface-open pores. These observations indicate that the extent of surface pore development cannot be predicted solely from density-based porosity measurements.

The apparent discrepancy originates from the fundamentally different principles of the analytical methods. Scanning electron microscopy (SEM) provides direct visualization of local surface morphology, pore opening, and internal pore architecture, whereas tapped-density-derived porosity represents a bulk density-derived parameter that reflects the combined contributions of intraparticle pores, interparticle voids, and particle packing characteristics [18,22]. Although *P*_corr_ partially accounts for the contribution of interparticle voids by subtracting the apparent porosity of the corresponding non-porous reference, it remains a density-derived comparative parameter and does not directly measure intraparticle pore volume, surface pore accessibility, or pore connectivity.

Accordingly, *P*_inc_ should be interpreted as a formulation-comparison parameter reflecting the additional porosity associated with the introduction of the porogen under the corresponding processing conditions, whereas *P*_corr_ provides a broader density-derived corrected porosity estimate relative to a non-porous reference. In PEG-PDLLA-containing composites, the observed porous morphology may therefore arise from the combined effects of porogen incorporation and PEG-PDLLA-associated changes in polymer organization and particle morphology. The magnitude of *P*_inc_ alone cannot distinguish these contributions or quantify the total internal pore volume.

These findings suggest that surface pore morphology and density-derived porosity characteristics should be evaluated independently when characterizing porous biodegradable microspheres. Surface-open pores may increase the accessibility of the microsphere surface to biological fluids and cells, thereby providing a greater accessible interface for initial cell attachment and cell–material or extracellular matrix interactions [19,20,22]. In contrast, density-derived porosity provides complementary information on the bulk porous characteristics of the microspheres, which may be associated with fluid transport and subsequent structural remodeling. Higher internal pore volume may also provide greater space for cell infiltration and tissue ingrowth when the pores are sufficiently interconnected, suggesting that surface accessibility and internal porous volume may contribute differently but complementarily to subsequent tissue integration. However, these potential biological effects were not directly evaluated in the present study and may also depend on pore size and connectivity, surface chemistry, degradation behavior, and mechanical properties. Therefore, the present structural characterization should be regarded as a basis for future biological evaluation rather than as direct evidence of enhanced cell infiltration or in vivo tissue integration.

Therefore, comprehensive characterization of porous biodegradable microspheres requires the combined evaluation of SEM morphology and density-based porosity measurements rather than reliance on either parameter alone. Importantly, *P*_corr_ and *P*_inc_ should be interpreted according to their respective calculation methods and should not be treated as equivalent measures of the total interparticle porous volume.

### 3.6. Micro-CT Analysis of Internal Structural Architecture

Micro-computed tomography (Micro-CT) was performed to qualitatively compare the internal architecture of PLLA microspheres and PLLA/PEG-PDLLA composite microspheres (Figure 6). Representative two-dimensional (2D) cross-sectional images and three-dimensional (3D) reconstructed images revealed distinct differences in the internal density distribution between the two formulations.

The PLLA microspheres exhibited a relatively homogeneous internal structure with finely dispersed low-density regions throughout the particles (Figure 6a,c). In contrast, the PLLA/PEG-PDLLA composite microspheres displayed a more heterogeneous internal density distribution, characterized by larger and more continuous low-density regions (Figure 6b,d). These observations suggest that PEG-PDLLA incorporation was associated with differences in both the surface morphology and the internal architecture of the microspheres.

The observed differences are consistent with the amphiphilic nature of PEG-PDLLA and may reflect polymer redistribution or phase-separation-related behavior during solvent evaporation [30,31]. However, the present Micro-CT analysis does not directly resolve PEG-rich and host-polymer-rich domains. The qualitative Micro-CT observations are in good agreement with the SEM results (Figure 5), which showed well-developed porous structures following PEG-PDLLA incorporation, and with the BET analysis, which showed an increase in nitrogen-accessible surface area. Although the spatial resolution of the present Micro-CT analysis does not permit quantitative evaluation of individual pore dimensions or pore connectivity, it provides complementary qualitative evidence of differences in the three-dimensional internal architecture between PLLA and PLLA/PEG-PDLLA microspheres. In addition, reliable quantitative segmentation requires optimized thresholding to distinguish intraparticle pores from the polymer matrix, interparticle spaces, and image noise. Further quantitative analysis of pore volume, pore connectivity, and open- and closed-pore fractions will require optimized image segmentation and thresholding procedures with higher-resolution datasets.

### 3.7. BET Analysis of Nitrogen-Accessible Surface and Pore Characteristics

Nitrogen adsorption–desorption analysis was performed to evaluate the nitrogen-accessible surface and pore characteristics of the microspheres. The BET specific surface area, total pore volume, and average pore diameter determined by the BJH method are summarized in Table 5.

To investigate the influence of particle size fractionation, porous PDLLA (R207S) microspheres before and after mesh fractionation were first compared. The unfractionated microspheres exhibited a relatively low BET surface area (0.977 m^2^ g^−1^) and total pore volume (0.00122 cm^3^ g^−1^), with an average BJH pore diameter of 24.61 nm. Following fractionation to the <33 μm size fraction, the BET surface area increased more than threefold to 3.131 m^2^ g^−1^, accompanied by a corresponding increase in total pore volume to 0.00512 cm^3^ g^−1^, whereas the average pore diameter decreased to 10.35 nm. These results indicate that particle-size fractionation increased the nitrogen-accessible surface area and pore volume while shifting the accessible pore structure toward smaller mesopores, consistent with the particle-size-dependent structural differences observed by the complementary analyses.

The increase in BET surface area may partly result from the greater external specific surface area associated with the smaller particle size; however, the concurrent increase in total pore volume and shift toward smaller nitrogen-accessible pores suggest that changes in the accessibility of the internal porous structure also contributed. The relative contributions of particle-size reduction and internal pore accessibility cannot be quantitatively separated from the present adsorption data, and the higher BET surface area of the <33 μm fraction should therefore be interpreted as a combined effect rather than as evidence of intrinsically greater mesoporosity alone.

The influence of PEG-PDLLA incorporation varied according to the physicochemical characteristics of the host polymer. In the PLLA system, the BET surface area was higher following PEG-PDLLA incorporation, increasing from 2.761 to 8.242 m^2^ g^−1^, while the total pore volume increased from 0.00611 to 0.01336 cm^3^ g^−1^. Simultaneously, the average BJH pore diameter decreased from 11.46 to 7.94 nm, suggesting an increased contribution of smaller nitrogen-accessible mesopores to the overall accessible pore structure. A similar but less pronounced trend was observed for PDLLA (R205S), where PEG-PDLLA increased the BET surface area from 4.869 to 5.263 m^2^ g^−1^ and the total pore volume from 0.00817 to 0.00905 cm^3^ g^−1^.

Among all formulations examined, the PDLLA (R207S)/PEG-PDLLA (5:5) composite exhibited the highest BET surface area (10.001 m^2^ g^−1^) and total pore volume (0.01652 cm^3^ g^−1^), indicating the greatest nitrogen-accessible specific surface area and pore volume among the formulations examined. In contrast, the PDLLA (R202S)/PEG-PDLLA (5:5) composite showed a comparatively lower BET surface area (3.516 m^2^ g^−1^) and pore volume (0.00563 cm^3^ g^−1^), suggesting that the morphological response associated with PEG-PDLLA incorporation varied with the molecular characteristics of the biodegradable polymer matrix.

The influence of PEG-PDLLA blending ratio was further evaluated using the PDLLA (R205S)-based system. Reducing the PEG-PDLLA content from a 5:5 to a 7:3 blend decreased the BET surface area from 5.263 to 3.393 m^2^ g^−1^, whereas the total pore volume changed only slightly (0.00905 to 0.00831 cm^3^ g^−1^). In contrast, the average pore diameter increased markedly from 8.69 to 17.61 nm, suggesting that the lower PEG-PDLLA content favored a pore structure with a lower accessible surface area and relatively larger nitrogen-accessible pores. These results indicate that the PEG-PDLLA composition influences not only the accessible surface area but also the pore-size characteristics of the nitrogen-accessible pore structure.

BET analysis primarily provides information on the specific surface area accessible to nitrogen adsorption, whereas BJH analysis provides an estimate of the accessible pore-size distribution within the applicable pore-size range. Whereas SEM provides direct morphological information on surface openings and internal pore architecture at the imaged locations, gas adsorption analysis reflects the nitrogen-accessible surface and pore structure that may not be directly resolved by SEM.

Consequently, microspheres exhibiting similar surface morphology may display substantially different BET surface areas depending on the accessibility of their internal pore surfaces. However, gas adsorption analysis does not independently quantify the total intraparticle pore volume or pore connectivity of the microspheres. The BET results obtained in this study are consistent with the SEM and Micro-CT observations, collectively suggesting that PEG-PDLLA incorporation and particle-size fractionation were associated with changes in the nitrogen-accessible porous structure of the biodegradable microspheres.

### 3.8. DSC Analysis of Thermal Properties and Their Relationship with Microsphere Morphology

Differential scanning calorimetry (DSC) was performed to investigate the thermal characteristics of the biodegradable polymers and to evaluate their possible relationship with porous structure formation during microsphere fabrication. The characteristic thermal transition temperatures of the representative polymers and PEG-PDLLA-containing composite microspheres are summarized in Table 6.

The amorphous PDLLA systems exhibited broad T_g_-related thermal transitions without distinct melting peaks, consistent with the predominantly amorphous character of PDLLA [10,11]. The T_g_-related transition increased from 47.7 °C for the low-molecular-weight PDLLA (R202S) to 55.9 °C for the high-molecular-weight PDLLA (R207S), suggesting reduced segmental mobility with increasing molecular weight. Incorporation of PEG-PDLLA into the R202S formulation shifted the T_g_-related transition to 43.0 °C, suggesting increased molecular mobility potentially associated with PEG-PDLLA incorporation. In contrast, no distinct T_g_-related transition was resolved for the R207S/PEG-PDLLA composite, whereas a characteristic PEG-domain melting transition appeared at 47.6 °C. This observation suggests that the PEG crystalline domains became thermally distinguishable from the broad T_g_-related response of the amorphous PDLLA matrix. These thermal characteristics are consistent with the amorphous PDLLA matrix and may be associated with the extensive internal pore development observed by SEM. However, the high corrected density-derived porosity index of PDLLA cannot be attributed to its amorphous character alone. The semicrystalline PLLA system exhibited a characteristic polymer melting temperature (T_m_) of 176.3 °C, consistent with its characteristic semicrystalline thermal behavior [10,11]. Following incorporation of PEG-PDLLA, the polymer melting temperature decreased slightly to 171.0 °C, while an additional PEG-domain melting transition was observed at 48.1 °C. These results indicate that the characteristic melting behavior of the semicrystalline PLLA matrix was retained after PEG-PDLLA incorporation, although a modest shift in T_m_ was observed. This interpretation agrees well with the SEM and Micro-CT observations, which showed well-developed porous structures and increased internal structural heterogeneity while maintaining spherical integrity.

Similarly, PCL exhibited a characteristic melting transition at 56.3 °C, which decreased only slightly to 55.6 °C after PEG-PDLLA incorporation. The minimal change in melting temperature indicates that the characteristic thermal transition of PCL was largely retained after PEG-PDLLA incorporation, consistent with its established semicrystalline nature [9]. A distinct PEG-domain melting transition could not be clearly resolved because it overlapped with the melting transition of the semicrystalline PCL matrix. These thermal characteristics, together with the SEM observations of relatively dense internal structures, suggest that the semicrystalline characteristics of PCL may contribute to its pore-formation behavior; however, the present DSC data do not establish crystallinity as the sole determinant of the limited pore development.

Overall, the DSC results indicate distinct thermal-transition behavior among the investigated host polymers and their PEG-PDLLA-containing composites. These thermal characteristics may contribute to polymer-dependent morphology development together with other factors such as molecular weight, chain mobility, solution behavior, and solidification kinetics. The amorphous PDLLA systems exhibited T_g_-related transitions without crystalline melting, consistent with greater segmental mobility relative to a semicrystalline polymer framework. In contrast, the semicrystalline PLLA and PCL systems retained their characteristic polymer melting transitions after PEG-PDLLA incorporation, indicating preservation of their characteristic thermal behavior. Because the degree of crystallinity was not quantitatively determined, these DSC results should not be interpreted as establishing a direct quantitative relationship between crystallinity and porosity.

Importantly, the incorporation of PEG-PDLLA produced only minor changes in the characteristic thermal transitions of the host polymers while introducing additional PEG-domain thermal transitions in polymer systems where the PEG crystalline domains could be clearly resolved. The relatively small shifts in the thermal transitions are considered primarily consistent with physical blending, altered chain packing, and changes in chain mobility associated with the PEG-PDLLA incorporation. However, partial intermolecular interactions between PEG-PDLLA and the host polymer chains cannot be excluded based on DSC alone. Therefore, the observed thermal shifts should be interpreted as changes in polymer chain mobility and packing rather than definitive evidence of specific intermolecular interactions. Such thermal behavior is consistent with the role of PEG-containing amphiphilic block copolymers in modifying the physical properties and phase behavior of biodegradable polymer matrices [30]. These findings support the interpretation of PEG-PDLLA as an amphiphilic morphology-modifying polymer component associated with changes in microsphere morphology and accessible porous structure. However, DSC does not directly demonstrate PEG-rich/host-polymer-rich phase separation, and alternative processes such as polymer redistribution during emulsion processing and washing may also contribute to the observed morphology. This interpretation is consistent with the complementary SEM, Micro-CT, BET, and porosity analyses, collectively suggesting that host-polymer physicochemical properties and PEG-PDLLA-associated morphology modification contribute to the observed structural differences among the biodegradable microspheres.

Residual DCM was not quantitatively determined in the present study, and therefore its potential plasticizing effect on the measured thermal transitions cannot be excluded. Accordingly, the DSC results were interpreted as comparative thermal characteristics among the investigated formulations rather than as direct evidence of differences in crystallinity or residual-solvent content.

### 3.9. Proposed Interpretation of Polymer-Dependent Porous Microsphere Formation

The experimental results obtained from SEM, density-derived porosity analysis, BET, Micro-CT, and DSC collectively suggest that porous microsphere formation is associated with the combined effects of polymer physicochemical properties, PEG-PDLLA-associated morphology modification, porogen-assisted pore formation, and particle size (Table 7 and Figure 7).

Rather than acting independently, these factors may collectively contribute to the final porous architecture of biodegradable microspheres. Among the investigated biodegradable polymers, PDLLA exhibited extensive internal pore development, consistent with its amorphous structure and relatively high chain mobility [10,11,15]. The absence of a semicrystalline framework may provide greater chain mobility during solvent evaporation and solidification, which may contribute to the development and preservation of internal porous features. In contrast, the semicrystalline PLLA matrix maintained spherical morphology while maintaining structural morphology [10,11,15]. PCL exhibited comparatively limited internal pore development under the present processing conditions [9,15]. Taken together, these observations suggest that polymer-dependent pore formation may reflect the combined effects of molecular characteristics, chain mobility, crystallinity-related chain packing, and solidification behavior rather than crystallinity alone. Because quantitative crystallinity was not determined in the present study, these relationships should be interpreted as formulation-dependent structural trends rather than as direct evidence of a causal relationship between crystallinity and pore formation.

PEG-PDLLA was associated with an additional morphology-modifying effect that differed from that observed with conventional porogen-assisted pore formation. Based on previous reports describing amphiphilic block-copolymer behavior and phase-separation-associated porous microsphere formation [16,30,31,34], the amphiphilic structure of PEG-PDLLA may contribute to changes in polymer distribution and interfacial organization during solvent evaporation, potentially contributing to the formation of surface-open porous structures even in the absence of an internal aqueous porogen. However, the present analyses do not directly demonstrate PEG-rich and host-polymer-rich phase-separated domains. Alternative processes, including the preferential migration of hydrophilic PEG segments toward the aqueous interface and partial removal or redistribution of PEG-containing domains during washing, may also contribute to the observed porous morphology and nitrogen-accessible surface area. Accordingly, PEG-PDLLA-mediated phase separation should be regarded as a proposed interpretation rather than a directly demonstrated mechanism.

When ammonium bicarbonate was simultaneously incorporated, decomposition of the porogen and the associated internal aqueous phase may have further contributed to pore formation, resulting in more developed highly porous architectures with increased accessible surface area [27,28]. The combined morphological effects of PEG-PDLLA incorporation and porogen-assisted pore formation were particularly evident in the PDLLA and PLLA systems, whereas the resulting morphology depended strongly on the physicochemical characteristics of the host polymer.

The present study suggests that the porogen may play two distinct roles during microsphere fabrication. In addition to contributing to internal pore generation, ammonium bicarbonate has been reported to influence emulsion behavior during porous microsphere fabrication [27,28]. In the present system, the dispersed internal aqueous phase may therefore have influenced W/O emulsion droplet behavior and the resulting microsphere size. However, the present study did not quantitatively separate the relative contributions of pore generation and changes in droplet behavior. Future mechanistic studies could therefore compare (i) a porogen-free polymer formulation, (ii) an NH_4_HCO_3_-containing formulation, (iii) a formulation containing a non-gas-generating pore-forming agent with comparable particle size and loading, and (iv) a gas-generating system designed to minimize solid-particle templating effects. Time-resolved measurement of droplet size distribution before and during solidification could be used to assess changes in droplet stability, whereas final microsphere size and circularity/sphericity, directly measured intraparticle pore volume, and surface-open pore density could be used to quantify the resulting morphological and pore-forming effects. Such controlled comparisons would help distinguish effects associated primarily with pore generation from those associated with changes in droplet stability and provide a framework for quantitative validation of the proposed dual-role interpretation. This behavior is consistent with the established effects of emulsion conditions and solvent evaporation on polymer microsphere formation [16,23,24,26].

Furthermore, particle size itself was associated with differences in pore preservation. The smaller microsphere fraction exhibited higher corrected density-derived porosity indices and nitrogen-accessible specific surface area; however, the underlying contribution of particle-size-dependent solvent diffusion and solidification kinetics was not directly measured. Smaller microspheres may undergo more rapid solvent exchange and solidification, which could contribute to preservation of porous morphology. These findings suggest that particle size may represent an additional processing-related factor associated with porous structure formation, rather than merely being a consequence of emulsification.

Based on these observations, porous microsphere formation may be interpreted within a framework comprising four complementary design factors: (i) intrinsic polymer properties, particularly molecular characteristics, chain mobility, and crystallinity-related chain packing; (ii) PEG-PDLLA-associated amphiphilic morphology modification; (iii) porogen-assisted internal pore generation and changes in emulsion behavior; (iv) particle-size-associated differences in solidification and pore preservation. Their proposed contributions are summarized in Table 7 and schematically illustrated in Figure 7.

Overall, this interpretative framework integrates the observed polymer-dependent morphological trends during solvent evaporation and may provide a basis for further investigation of formulation strategies for tailoring biodegradable porous microspheres with controlled internal architecture and pore accessibility. The proposed pathways should be regarded as interpretations of the observed structural trends rather than directly resolved molecular-scale mechanisms.

These formulation considerations may be broadly applicable to biodegradable polymer systems intended for tissue engineering, regenerative medicine, injectable biomaterials, and controlled drug-delivery applications.

## 4. Conclusions

In this study, biodegradable porous microspheres based on PDLLA, PLLA, PCL, and amphiphilic PEG-PDLLA were systematically investigated to evaluate the relationships between polymer physicochemical properties and porous structure formation and morphology development.

The results indicated that the observed porous microsphere morphology was associated not only with porogen incorporation but also with polymer molecular characteristics, chain mobility, crystallinity-related characteristics, PEG-PDLLA-associated amphiphilic morphology modification, and solidification behavior. Amorphous PDLLA exhibited the highest corrected density-derived porosity index and extensive internal pore formation, whereas semicrystalline PLLA exhibited moderate pore development while maintaining spherical morphology. In contrast, PCL showed limited internal pore development and comparatively dense structures under the present fabrication conditions.

PEG-PDLLA was associated with changes in microsphere morphology rather than acting solely as a conventional porogen. Its incorporation was associated with surface-open pore formation and changes in the accessible porous structure in a polymer-dependent manner. When combined with an internal aqueous porogen, more developed porous structures and increased accessible surface area were observed in the PLLA and PDLLA systems. In addition, blending PDLLA grades with different molecular weights was associated with differences in microsphere morphology and pore preservation, suggesting that molecular weight may represent an additional formulation parameter associated with microsphere morphology.

The complementary analyses using SEM, Micro-CT, BET, DSC, and density-based porosity measurements indicated that surface morphology, internal pore architecture, nitrogen-accessible pore structure, and thermal properties represent complementary structural descriptors rather than interchangeable measures of porosity. Their combined evaluation therefore provides a more comprehensive basis for characterizing formulation-dependent morphological differences in biodegradable porous microspheres. Because independently prepared replicate batches were not available for all formulations, the observed differences should be interpreted as descriptive experimental trends rather than statistically established effects.

Overall, this study provides insight into polymer-property-dependent morphological trends during solvent evaporation. The findings provide a basis for further investigation of formulation strategies for tailoring biodegradable porous microspheres with controlled pore architecture, surface accessibility, and structural stability, with potential applications in regenerative fillers, injectable biomaterials, tissue-engineering scaffolds, cell-interactive carriers, and controlled drug-delivery systems.

## 5. Patents

Application no. 10-2026-0111801 (Republic of Korea), filed by VAIM Co., Ltd.

## Figures and Tables

**Figure 1:**
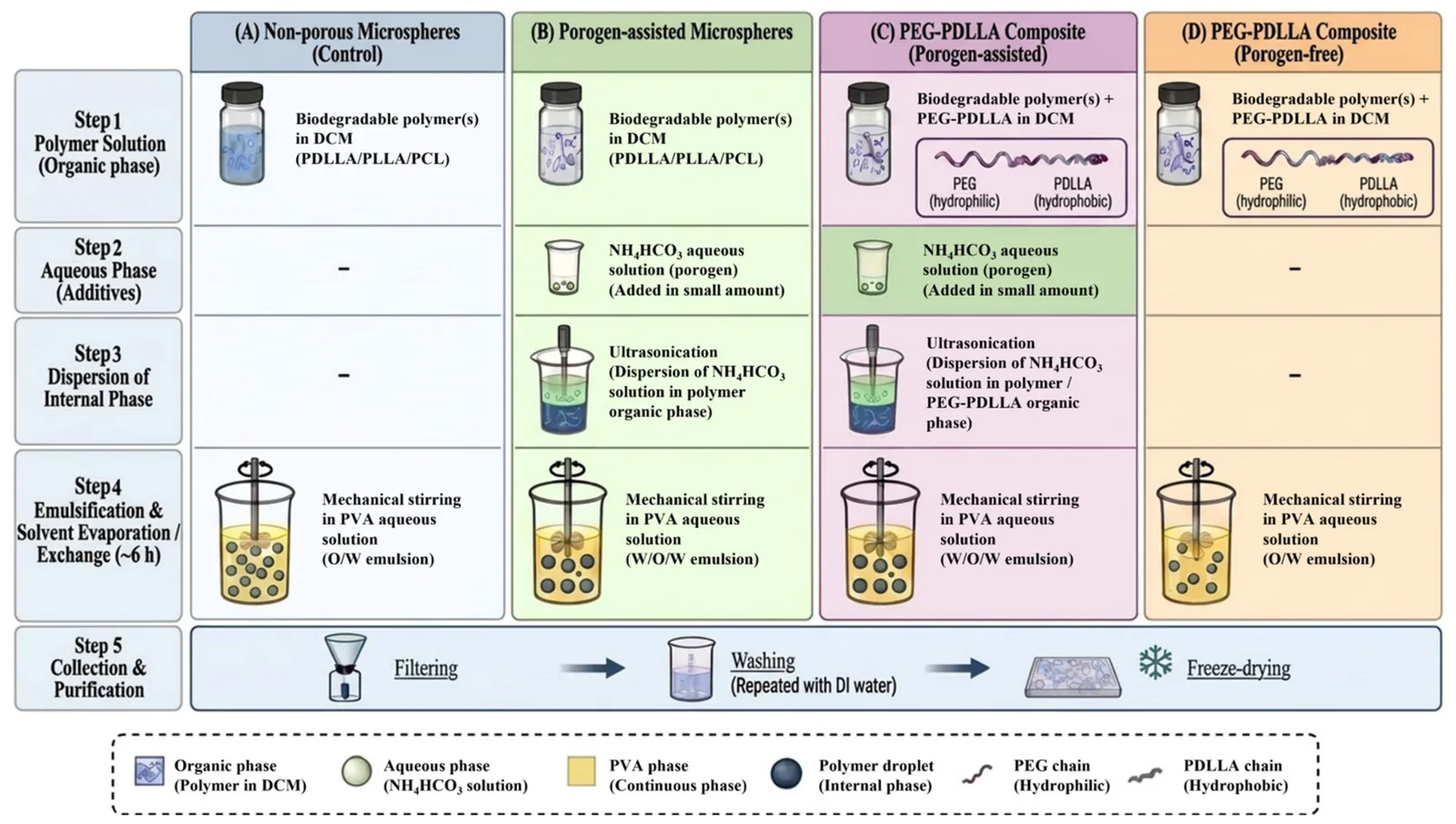
Four preparation routes were used to fabricate biodegradable microspheres with different PEG-PDLLA and porogen conditions. Non-porous microspheres (**A**), porogen-assisted porous microspheres (**B**), PEG-PDLLA composite porous microspheres prepared with porogen (**C**), and PEG-PDLLA composite microspheres prepared without porogen (**D**) were fabricated by O/W or W/O/W solvent evaporation, followed by filtration, washing, and freeze-drying before physicochemical characterization.

**Figure 2:**
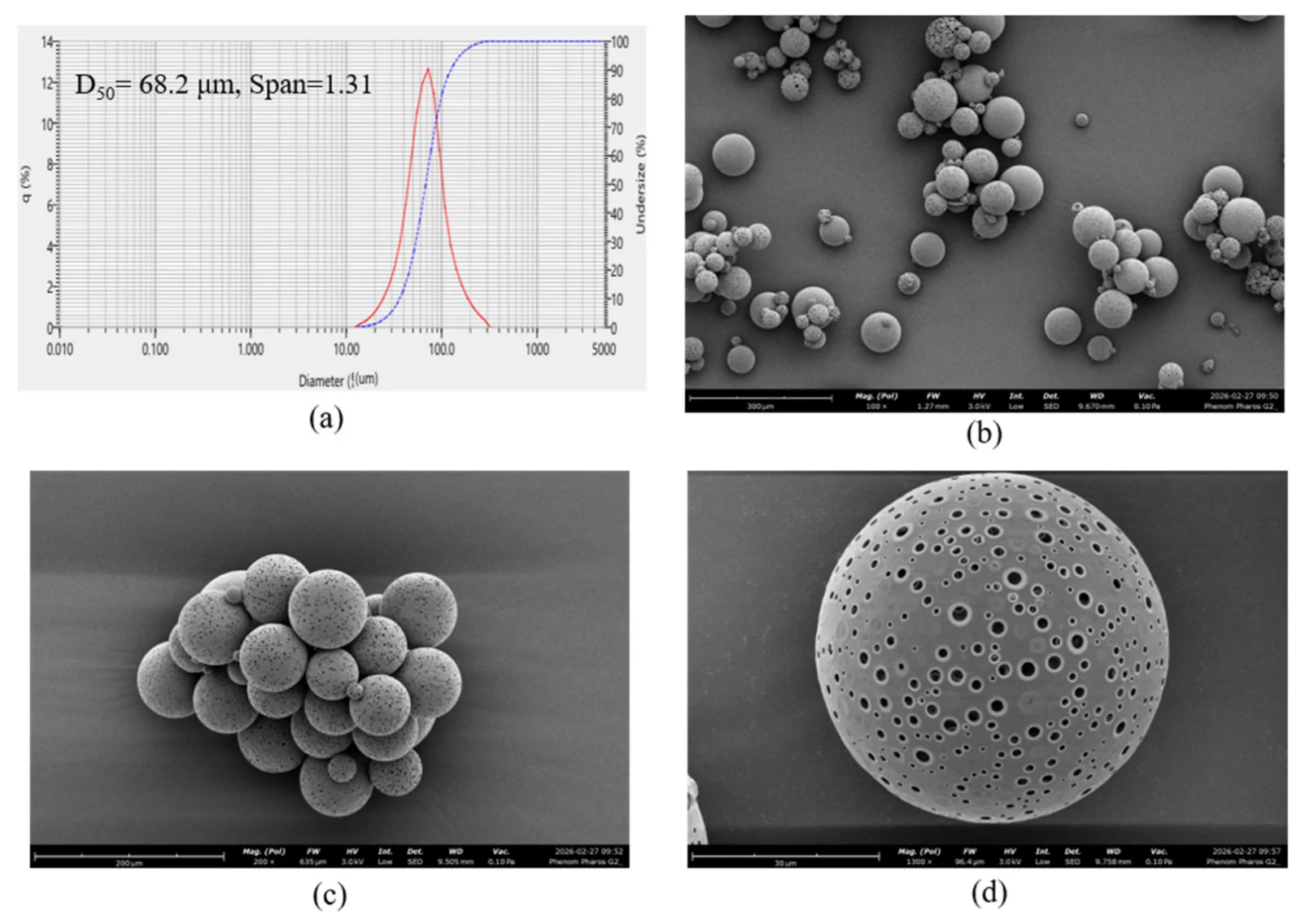
(**a**) Particle size distribution of the as-prepared porous PDLLA microspheres measured by laser diffraction, showing a median particle diameter (D_50_) of 68.2 μm and a Span value of 1.31. The red line represents the differential volume-based particle size distribution, and the blue line represents the cumulative volume distribution. (**b**–**d**) Representative SEM images acquired at increasing magnifications. The microspheres exhibited a well-defined spherical morphology, and the higher-magnification image (**d**) revealed numerous surface-open pores distributed over the particle surface. No apparent particle collapse, cracking, or severe deformation was observed.

**Figure 3:**
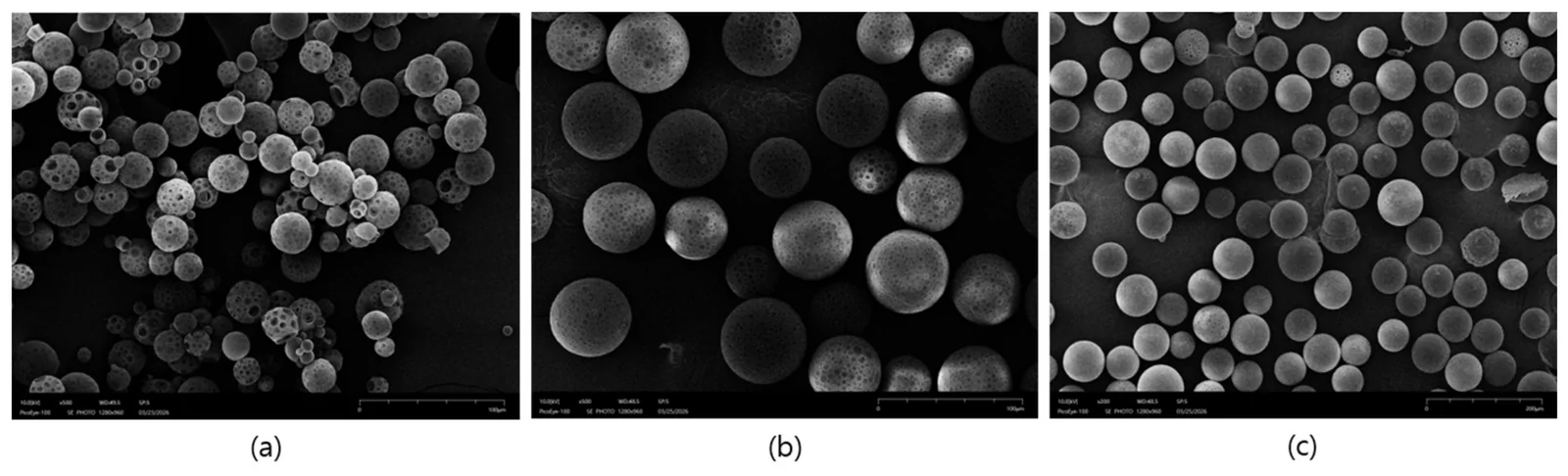
Representative SEM images of particle size-fractionated porous PDLLA microspheres. (**a**) <33 μm, (**b**) 33–60 μm, and (**c**) >60 μm fractions obtained using the suspended planar mesh fractionation process. All fractions exhibited spherical morphology with surface-open pores distributed over the particle surfaces, with no apparent particle deformation, fracture, or pore collapse.

**Figure 4:**
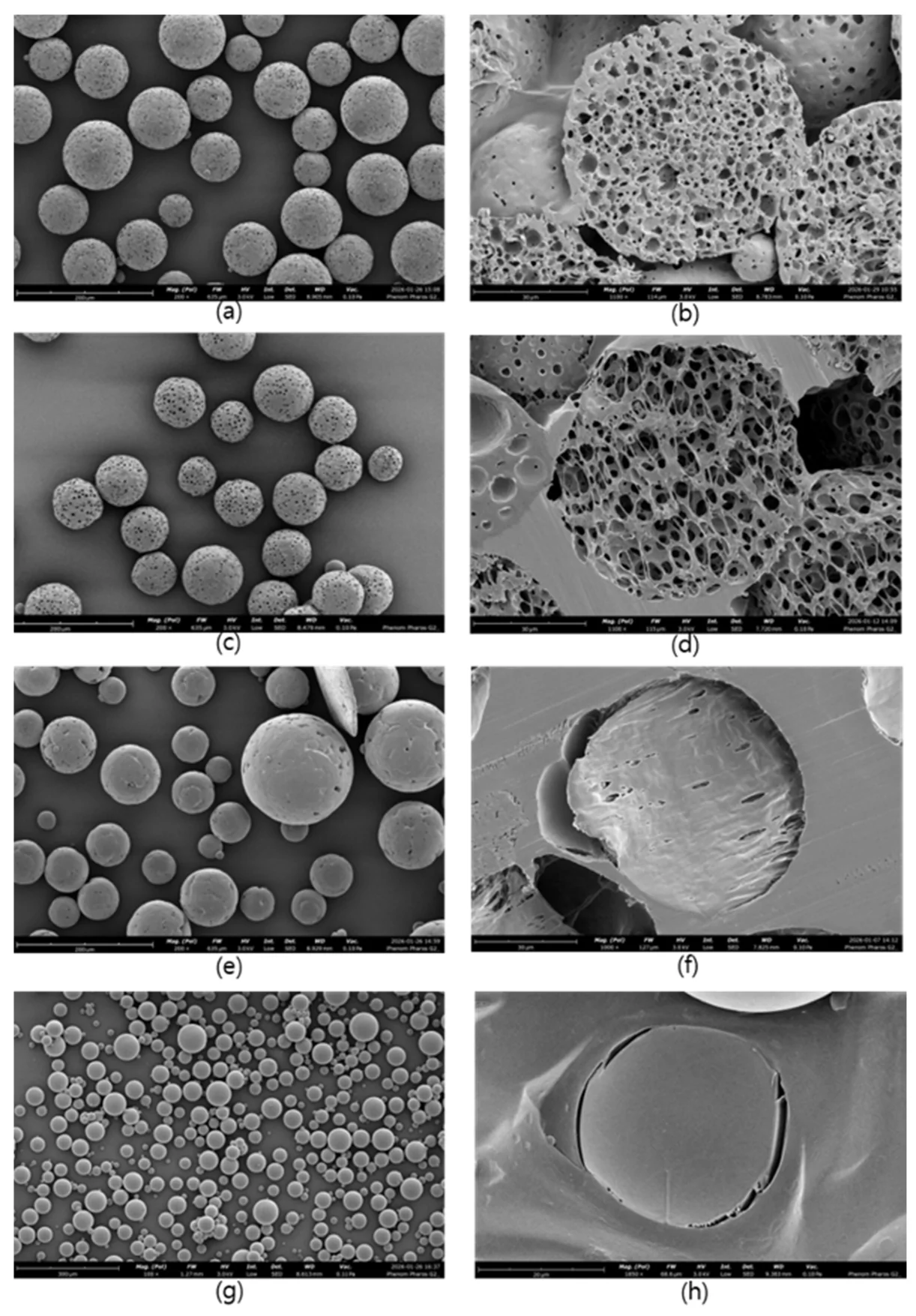
Representative SEM images showing the surface morphology (**left column**) and cross-sectional pore architecture (**right column**) of biodegradable microspheres prepared from different polymer systems. (**a**,**b**) PLLA (L206S, porogen-assisted), (**c**,**d**) PDLLA (R207S, porogen-assisted), (**e**,**f**) PCL (C209, porogen-assisted), and (**g**,**h**) PDLLA (R207S, porogen-free). Porogen-assisted PDLLA exhibited extensive internal pore development, whereas PLLA showed moderate internal pore development and PCL exhibited a predominantly dense internal structure with limited pores. Porogen-free PDLLA maintained spherical morphology but exhibited a predominantly dense internal structure with minimal pore development.

**Figure 5:**
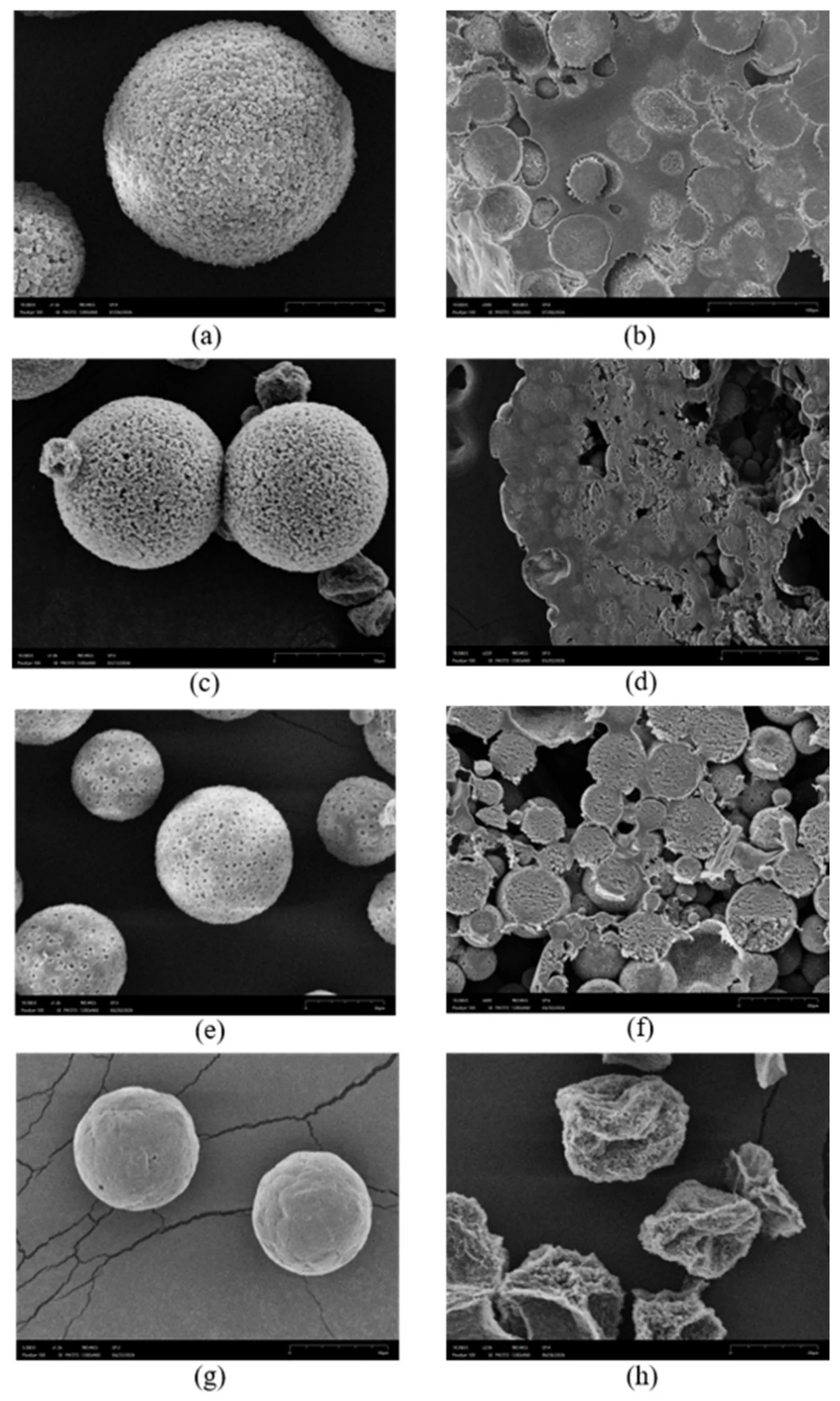
Representative SEM images showing the surface morphology and internal pore architecture of PEG-PDLLA-containing composite microspheres prepared under porogen-assisted and porogen-free conditions. (**a**,**b**) PDLLA (R205S)/PEG-PDLLA (5:5) prepared under porogen-free conditions; (**c**,**d**) PLLA/PEG-PDLLA (5:5) prepared under porogen-assisted conditions; (**e**,**f**) PLLA/PEG-PDLLA (5:5) prepared under porogen-free conditions; (**g**) PCL/PEG-PDLLA (7:3) prepared under porogen-free conditions; (**h**) PEG-PDLLA prepared without a host biodegradable polymer under porogen-free conditions. Cross-sectional images show the internal pore architecture, whereas surface images show external morphology and overall particle shape.

**Figure 6:**
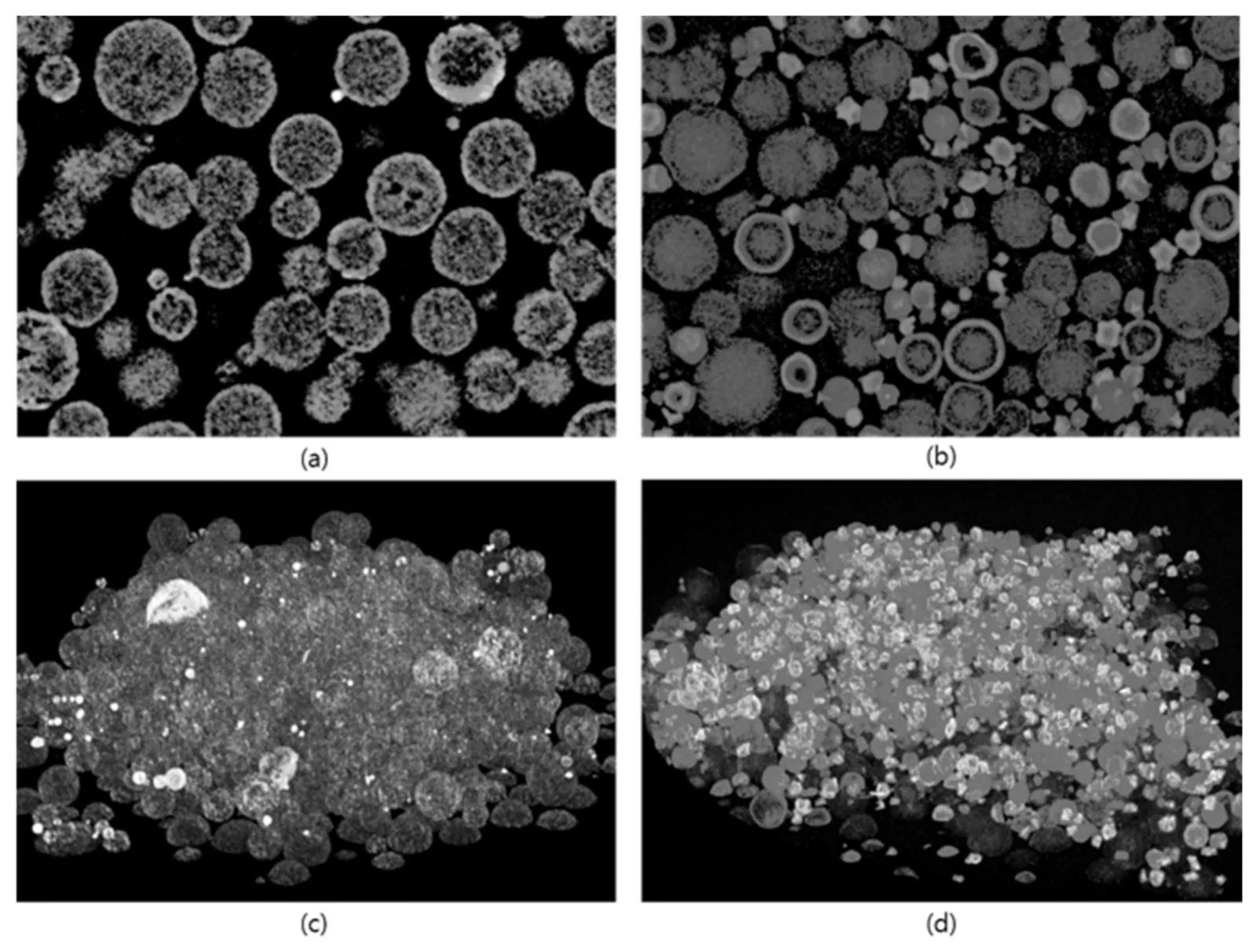
Representative micro-computed tomography (Micro-CT) images comparing the internal architecture of PLLA and PLLA/PEG-PDLLA composite microspheres. (**a**,**b**) Two-dimensional (2D) cross-sectional images and (**c**,**d**) three-dimensional (3D) reconstructed images of PLLA (**left**) and PLLA/PEG-PDLLA (**right**) microspheres. The PLLA/PEG-PDLLA composite microspheres exhibited a more heterogeneous internal density distribution with more prominent low-density regions than the PLLA microspheres, indicating qualitative differences in the internal architecture between the two formulations.

**Figure 7:**
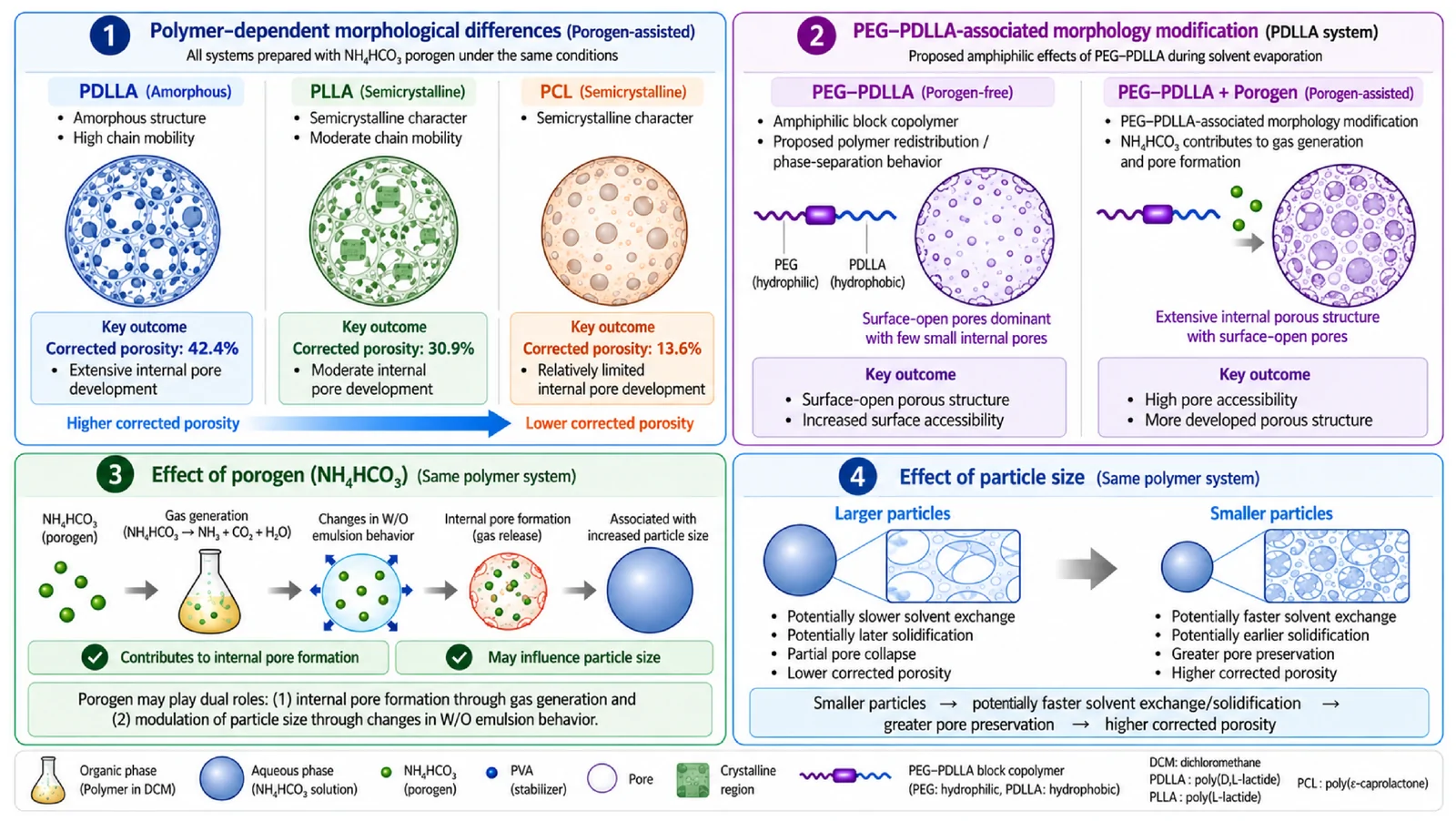
Proposed framework for polymer-property-dependent morphological development of biodegradable porous microspheres. Polymer molecular characteristics, chain mobility, crystallinity-related chain packing, PEG-PDLLA-associated amphiphilic morphology modification, porogen-assisted pore formation, and particle-size-associated solidification behavior may collectively contribute to pore formation and final microsphere morphology. PEG-PDLLA-associated morphology modification may involve polymer redistribution or phase-separation-related behavior, while the porogen may contribute to both internal pore formation and changes in emulsion behavior. Particle-size-dependent solvent exchange and solidification are also proposed to contribute to pore preservation. The illustrated pathways represent proposed interpretations of the observed morphological trends rather than directly resolved molecular-scale mechanisms.

**Table 1:** Commercial biodegradable polymers used in this study and their physicochemical characteristics.

Polymer	Grade	Nominal Molecular Weight (kDa)	Polymer Characteristic
PDLLA	R202S	16	Amorphous
	R205S	63	
	R207S	190	
PLLA	L206S	91	Semicrystalline
PCL	C209	70	Semicrystalline
mPEG-DLPLA (PEG-PDLLA)	DL1005MP1	23.8	Amphiphilic diblock copolymer

Abbreviations: PDLLA, poly(D,L-lactide); PLLA, poly(L-lactide); PCL, poly(ε-caprolactone); mPEG-DLPLA, methoxy poly(ethylene glycol)-block-poly(D,L-lactide) (hereafter referred to as PEG-PDLLA). The nominal molecular weights were provided by the respective manufacturers according to the product specifications. Polymer characteristics, including amorphous or semicrystalline classification, were based on manufacturer information and/or literature data and do not represent quantitative crystallinity measurements performed in this study.

**Table 2:** Particle size distribution and corrected density-derived porosity index of size-fractionated PDLLA porous microspheres.

Fraction	Mesh Classification	D_10_ (μm)	D_50_ (μm)	D_90_ (μm)	Span	Corrected Density-Derived Porosity Index (%)
F1	<33 μm	18.7	28.7	41.6	0.80	50.5
F2	33–60 μm	35.5	46.6	62.1	0.57	42.4
F3	>60 μm	64.4	84.3	118.8	0.65	41.9

Span was calculated as (D_90_ − D_10_)/D_50_. The corrected density-derived porosity index was calculated to Equation (3) and was used as a comparative density-derived parameter rather than as a direct measure of absolute intraparticle porosity.

**Table 3:** Particle size distribution and density-derived porosity characteristics of porogen-assisted and porogen-free microspheres according to polymer type and molecular weight.

Polymer System	Grade	Mw (kDa)	Preparation	D_50_ (μm)	Span	Apparent Porosity (%)	Corrected Density-Derived Porosity Index (%)
PLLA	L206S	91	Porogen-assisted	50.2	0.89	79.6	30.9
			Porogen-free	34.2	0.85	48.7	-
PDLLA	R202S	16	Porogen-assisted	39.3	0.72	64.7	16.9
			Porogen-free	29.1	0.79	47.8	-
	R205S	63	Porogen-assisted	47.5	0.92	87.8	30.7
			Porogen-free	34.1	0.84	57.1	-
	R207S	190	Porogen-assisted	68.1	1.31	86.4	42.4
			Porogen-free	45.1	0.76	44	-
PCL	C209	70	Porogen-assisted	60.4	0.97	66.8	13.6
			Porogen-free	39.3	0.89	53.2	-

Span was calculated as (D_90_ − D_10_)/D_50_. The corrected density-derived porosity index was calculated as the difference between the apparent porosities of the corresponding porogen-assisted and porogen-free microspheres according to Equation (3).

**Table 4:** Morphological characteristics, particle size distribution, and density-derived porosity parameters of biodegradable microspheres according to polymer composition, PEG-PDLLA incorporation, and porogen incorparation.

Polymer System	Preparation	D_50_ (μm)	Span	Apparent Porosity (%)	Porogen- Induced Incremental Porosity (%)
PLLA/	Porogen-assisted	78.9	0.97	90.1	8.6
PEG-PDLLA (5:5)	Porogen-free	50.8	1.16	83.3	
PLLA/	Porogen-assisted	80.8	0.94	90.8	28.2
PEG-PDLLA (7:3)	Porogen-free	54.7	1.56	62.6	
PDLLA (R202S)/	Porogen-assisted	49.3	1.13	95.1	N.D.
PEG-PDLLA (5:5)	Porogen-free	Irregular aggregates			
PDLLA (R205S)/	Porogen-assisted	84.7	0.92	91.9	12.5
PEG-PDLLA (5:5)	Porogen-free	81.8	0.78	79.4	
PDLLA (R205S)/	Porogen-assisted	69.7	1.41	94.5	15.9
PEG-PDLLA (7:3)	Porogen-free	43.3	0.88	78.6	
PCL/	Porogen-assisted	Irregular aggregates			N.D.
PEG-PDLLA (5:5)	Porogen-free	32.9	2.14	63	
PCL/PEG-PDLLA	Porogen-assisted	56.8	0.90	72.1	19.8
(7:3)	Porogen-free	47.5	0.81	52.3	
PDLLA (R202S)/	Porogen-assisted	71.6	0.83	90.8	32.7 ^†^
PDLLA (R207S) (5:5)	Porogen-free	42.4	0.79	58.1	
PEG-PDLLA only	Porogen-assisted	69.7	1.16	83.1	N.D.
	Porogen-free	No recoverable microspheres			

Span = (D_90_ − D_10_)/D_50_. N.D., not determined because recoverable spherical microspheres were not obtained for porosity determination. For irregular aggregates, D_50_ values represent equivalent particle diameters measured by laser diffraction and do not indicate the diameter of spherical microspheres. Values in the “Porogen-induced incremental porosity” column represent the porogen-induced incremental porosity (*P*_inc_) calculated as in Equation (4), except for the row marked ^†^ (PDLLA (R202S)/PDLLA (R207S) blend), which contains no PEG-PDLLA. For this formulation, the corresponding porogen-free formulation was used as the non-porous reference, and the tabulated value represents the corrected density-derived porosity index (*P*_corr_) calculated as in Equation (3), consistent with Table 3.

**Table 5:** BET surface area, BJH adsorption pore volume, and BJH pore diameter of porous microspheres according to particle size fractionation, PEG-PDLLA incorporation, and polymer blending ratio.

Polymer Composition	BET Surface Area (m^2^/g)	Total Pore Volume (cm^3^/g)	Average Pore Size (BJH, nm)
Effect of particle size fractionation			
PDLLA (R207S)	0.977	0.00122	24.61
PDLLA (R207S, <33 μm fraction)	3.131	0.00512	10.35
Effect of PEG-PDLLA incorporation			
PLLA (L206S)	2.761	0.00611	11.46
PLLA/PEG-PDLLA (5:5)	8.242	0.01336	7.94
PDLLA (R205S)	4.869	0.00817	9.37
PDLLA (R205S)/PEG-PDLLA (5:5)	5.263	0.00905	8.69
PDLLA (R207S)/PEG-PDLLA (5:5)	10.001	0.01652	9.17
PDLLA (R202S)/PEG-PDLLA (5:5)	3.516	0.00563	7.73
Effect of PEG-PDLLA blending ratio			
PDLLA (R205S)/PEG-PDLLA (5:5)	5.263	0.00905	8.69
PDLLA (R205S)/PEG-PDLLA (7:3)	3.393	0.00831	17.61

BET surface area was determined using the Brunauer–Emmett–Teller (BET) method [37]. BJH adsorption pore volume and average pore diameter were calculated from the adsorption branch using the Barrett–Joyner–Halenda (BJH) method [38].

**Table 6:** Characteristic thermal transition temperatures of representative biodegradable polymers and PEG-PDLLA composite microspheres determined by differential scanning calorimetry (DSC).

Sample	T_g_-Related Transition (°C)	PEG-Domain T_m_ (°C)	Polymer T_m_ (°C)
PEG-PDLLA	-	49.1	-
PDLLA (R202S)	47.7	-	-
PDLLA (R202S)/PEG-PDLLA (5:5)	43.0	- *	-
PDLLA (R207S)	55.9		-
PDLLA (R207S)/PEG-PDLLA (5:5)	-	47.6	-
PLLA (L206S)	-	-	176.3
PLLA/PEG-PDLLA (5:5)	-	48.1	171.0
PCL (C209)	-	-	56.3
PCL/PEG-PDLLA (5:5)	-	- *	55.6

* The PEG-domain melting transition could not be clearly resolved because of overlap with the T_g_-related thermal response of the PDLLA matrix in the PDLLA (R202S)/PEG-PDLLA composite, or with the melting transition of the PCL matrix in the PCL/PEG-PDLLA composite.

**Table 7:** Summary of factors associated with pore formation and morphology development in biodegradable microspheres.

Polymer System	Key Material/ Process Factor	Observed/Proposed Effect on Morphology	Representative Morphology
PDLLA	Amorphous polymer	Associated with extensive internal pore development, potentially related to greater chain mobility	Highly porous microspheres with extensive internal pore development
PLLA	Semicrystalline polymer	Maintains particle integrity while permitting pore development	Spherical porous microspheres with stable morphology
PCL	Semicrystalline polymer	Associated with comparatively limited internal pore development under the investigated conditions	Relatively dense internal structure
PDLLA R202S/ 207S blend	Molecular-weight combination	Associated with improved spherical morphology and preservation of porous architecture	Uniform porous microspheres
PDLLA/ PEG-PDLLA	Amphiphilic morphology modifier	Associated with surface-open pore formation and changes in accessible porous structure	Surface-open and internally porous microspheres
PLLA/ PEG-PDLLA	Semicrystalline matrix + amphiphilic morphology modifier	Maintains spherical integrity while modifying internal porous structure	Porous microspheres with internal low- density regions

## Data Availability

The raw data supporting the conclusions of this article will be made available by the authors upon reasonable request.
